# A conserved set of mutations for stabilizing soluble envelope protein dimers from dengue and Zika viruses to advance the development of subunit vaccines

**DOI:** 10.1016/j.jbc.2022.102079

**Published:** 2022-05-26

**Authors:** Thanh T.N. Phan, Matthew G. Hvasta, Stephan T. Kudlacek, Devina J. Thiono, Ashutosh Tripathy, Nathan I. Nicely, Aravinda M. de Silva, Brian Kuhlman

**Affiliations:** 1 Department of Biochemistry and Biophysics, University of North Carolina at Chapel Hill, Chapel Hill, North Carolina, USA; 2 Department of Microbiology and Immunology, University of North Carolina at Chapel Hill, Chapel Hill, North Carolina, USA; 3 Department of Pharmacology, University of North Carolina at Chapel Hill, Chapel Hill, North Carolina, USA

**Keywords:** antigen presentation, protein design, Rosetta, protein stability, vaccine development, dengue virus, Zika virus, flavivirus, soluble E dimer, antibody binding, Ab, antibody, DENV, dengue virus, E, envelope protein, EDE, E-dimer epitope, IgG, immunoglobulin G, mAb, monoclonal antibody, MM, molar mass, nano-DSF, nano differential scanning fluorimetry, SC, stabilizing combination, sE, soluble E protein, SEC–MALS, size-exclusion chromatography coupled with multiangle light scattering experiment, ZIKV, Zika virus

## Abstract

Dengue viruses (DENV serotypes 1–4) and Zika virus (ZIKV) are related flaviviruses that continue to be a public health concern, infecting hundreds of millions of people annually. The traditional live-attenuated virus vaccine approach has been challenging for the four DENV serotypes because of the need to achieve balanced replication of four independent vaccine components. Subunit vaccines represent an alternative approach that may circumvent problems inherent with live-attenuated DENV vaccines. In mature virus particles, the envelope (E) protein forms a homodimer that covers the surface of the virus and is the major target of neutralizing antibodies. Many neutralizing antibodies bind to quaternary epitopes that span across both E proteins in the homodimer. For soluble E (sE) protein to be a viable subunit vaccine, the antigens should be easy to produce and retain quaternary epitopes recognized by neutralizing antibodies. However, WT sE proteins are primarily monomeric at conditions relevant for vaccination and exhibit low expression yields. Previously, we identified amino acid mutations that stabilize the sE homodimer from DENV2 and dramatically raise expression yields. Here, we tested whether these same mutations raise the stability of sE from other DENV serotypes and ZIKV. We show that the mutations raise thermostability for sE from all the viruses, increase production yields from 4-fold to 250-fold, stabilize the homodimer, and promote binding to dimer-specific neutralizing antibodies. Our findings suggest that these sE variants could be valuable resources in the efforts to develop effective subunit vaccines for DENV serotypes 1 to 4 and ZIKV.

Dengue virus (DENV) and Zika virus (ZIKV) are mosquito-borne flaviviruses that infect over 350 million people per year in tropical and subtropical regions around the world (1). Currently, there is no Food and Drug Administration–approved vaccine for ZIKV, and development of a vaccine for DENVs has been challenging because of the presence of four serotypes (DENV1–4) and the potential for DENV vaccines to stimulate antibodies that enhance WT DENV replication and disease severity. The leading live-attenuated tetravalent DENV vaccines designed to induce simultaneous immunity to the four serotypes have been plagued by imbalanced replication and variable efficacy depending on the DENV serotype and the DENV immune status prior to vaccination (2). Dengvaxia, a tetravalent vaccine developed by Sanofi Pasteur, was efficacious in children who had pre-existing immunity from WT DENV infections. In children with no pre-existing immunity, the vaccine increased the risk of severe dengue disease compared with children who received a placebo (2). As a result, there continues to be a strong need for safe vaccines that can provide balanced and long-lasting immunity against DENVs and ZIKV.

As members of the Flaviviridae family, the four DENV serotypes and ZIKV share common structural features. Flaviviruses are enveloped viruses with 180 copies of an integral membrane glycoprotein, termed the envelope (E) protein that forms a smooth protein coat with icosahedral symmetry covering the outer surface of the mature infectious virus (3). The E protein consists of three β-barrel domains: EDI, EDII, and EDIII. EDII contains a highly conserved epitope responsible for fusion of the virus to host membrane (4, 5), whereas EDIII is believed to be responsible for cellular adhesion and viral uptake by cell receptors (6, 7). On the surface of the virus, E proteins form homodimers, and groups of three homodimers interact to form higher order structures designated as “rafts.” Thirty rafts are packed in a herringbone pattern, creating a protein coat with icosahedral symmetry. Serum and B-cell analysis of infected patients has shown that the E protein is the primary target for antibodies that neutralize DENV and ZIKV (8, 9). Epitope mapping has revealed that many neutralizing antibodies, including potent type–specific monoclonal antibodies (mAbs) 2D22 (DENV2, (10, 11)) and G9E (ZIKV, (12)), bind to quaternary epitopes that span across two or more E monomers on the viral surface. The E-dimer epitope (EDE) is particularly interesting because some antibodies directed against it can neutralize all four DENV serotypes as well as ZIKV (13, 14, 15, 16). The EDE is located at the interface formed between EDII from one chain of the E dimer and EDI and EDIII from the other chain of the dimer (13, 15, 17).

Subunit vaccines based on the E protein offer an alternative vaccine strategy to live-attenuated viruses for DENV and ZIKV. This approach bypasses the challenge of uneven viral replication observed with live-attenuated vaccines and may provide a safer approach. Investigators have completed preclinical and phase I clinical trial with a tetravalent formulation of the ectodomain of E (DEN-80E–V180) (18, 19). While the antigens induced modest levels of neutralizing antibodies, poor efficacy in animal studies and waning immunity in humans have discouraged further development of this vaccine. Previous studies have shown that DENV serotypes 1 to 4 and ZIKV WT soluble E (sE) proteins have moderate thermostability and weak homodimer affinity at 37 °C (13, 20, 21). The estimated dissociation constants for WT sE dimers are in the micromolar range, which indicates that sE would be primarily monomeric under vaccination conditions. In addition, EDE and type-specific dimer antibodies have weak affinity for the WT sE proteins at physiological temperature (20, 21), indicative of low levels of E dimers in solution at 37 °C. Mice vaccinated with DENV2 WT sE elicited high levels of antibodies that are monomer specific and lower levels of dimer-specific antibodies (22). These results point toward a disadvantage in using WT sE as a subunit vaccine: loss of the region that is necessary to elicit important classes of potently neutralizing antibodies.

In a recently published study, we used the molecular modeling software Rosetta (Rosetta Commons) to identify amino acid mutations that stabilize the sE dimer from DENV2 and increased production yields by over 50-fold (Fig. 1*A*) (22). These mutations are located in clusters that are spread throughout the E protein: in the core of EDI (mutation set called PM4), in the hinge region between EDI and EDII (mutation set called U6), at the central αB helix in EDII (mutation set called I2), and in the fusion loop of EDII (mutation set called I8, (22)). I2 includes two mutations, A259W and T262R, that stabilize the E dimer by making pi–cation interactions with the symmetrical mutations on the other chain of the homodimer (Fig. 1). I8 consists of a single mutation, G106D, that forms new electrostatic interactions at the interface between EDII from one chain of the homodimer and EDIII from the other chain of the homodimer. Interestingly, U6 contains two mutations, T280P and F279W, that are not located at the homodimer interface but still stabilize the dimer. The U6 mutations are located in a flexible hinge that connects EDII and EDI and may stabilize the dimer by locking the hinge in a conformation that favors dimer formation (22). The PM4 mutations, S29K, T33V, and A35M, are located in the core of EDI and do not stabilize the formation of the DENV2 E dimer, but they raise the thermal unfolding temperature of DENV2 sE by more than 10 °C. Combining the four mutation sets results in a DENV2 sE variant that remains a dimer at concentrations below 1 nM and has a temperature of unfolding (*T*_m_) that is 14.9 °C higher than the WT protein. Mice immunized with this stabilized dimer elicited a population of dimer-specific binding antibodies that were not observed in mice immunized with WT DENV2 E protein. Moreover, the stable dimer elicited higher levels of DENV2-neutralizing antibodies compared with mice immunized with WT sE antigen (22).

**Figure 1:**
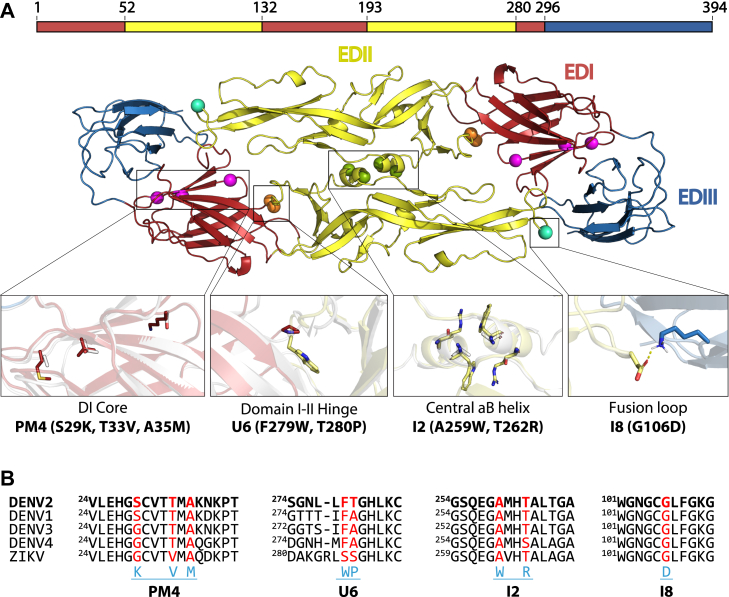
**Stabilizing mutations from DENV2 sE at conserved regions on the E protein.***A*, depiction of previously characterized mutations (*spheres*) in DENV2 sE with PM4 (*magenta*), U6 (*orange*), I2 (*green*), and I8 (*cyan*) mutation set outlined. Zoomed in panels show alignments between WT protein (*white*) and mutations (colored according to domains) within each set. PM4 and I8 structures are obtained from Rosetta models, whereas U6 and I2 are from crystal structure of DENV2 SC10 (I2-I8-U6). *B*, multiple sequence alignment of sE proteins from DENV1–4 and ZIKV using the WHO strains, showing ±5 residues in linear sequence from each mutation; DENV2 in *bold* as a reference. *Red residues* indicate positions of the mutations, and *blue amino acids* show the substitutions in each set at corresponding positions. All mutation sets are located in conserved regions of the E protein, with U6 being the most variable. DENV2, dengue virus serotype 2; E, envelope protein; SC, stabilizing combination; sE, soluble E protein; WHO, World Health Organization; ZIKV, Zika virus.

Here, we test if the mutations identified for DENV2 also stabilize the sE dimers from DENV1, DENV3, DENV4, and ZIKV. We find that the mutations improve production yields, thermostability, and dimer affinity, although in some cases, the increases are smaller than observed for DENV2. The stabilized E proteins also show high binding to EDE and type-specific mAbs at 37 °C, unlike their WT counterparts. These designed sE variants are promising candidates for a tetravalent subunit vaccine that can elicit antibodies targeting quaternary epitopes, including EDE antibodies.

## Results

### Rosetta-stabilizing mutations are in conserved regions of sE

We first examined a multiple sequence alignment of sE from the four DENV serotypes and ZIKV to see if the residues that were mutated to stabilize DENV2 sE are conserved in the other proteins (Fig. 1*B*). Overall, all four mutation sets are in highly conserved regions of sE. The residues mutated at the central αB helix (I2), in the core of EDI (PM4), and in the fusion loop (I8) are identical in all five proteins except that there is a Thr to Ser mutation in DENV4 at position 262 at the αB helix and a Thr to Val mutation in ZIKV at position 33 in the core of EDI. The two residues mutated in the hinge region between EDI and EDII (U6) are similar in the four DENV serotypes (Phe-Ala or Phe-Thr) but are Ser residues in ZIKV. We also examined the structure of sE to see if the residues proximal to the mutation sites are conserved. Aside from two residues located near the EDI and EDII hinge (U6), the residues neighboring the mutations are highly similar (Fig. S1). The high conservation at each mutation site as well as surrounding residues led us to hypothesize that the stabilizing mutations discovered in DENV2 sE would have similar effects when applied to the other DENV serotypes and ZIKV.

### Rosetta-stabilizing mutations increase protein production yields

**Figure 2:**
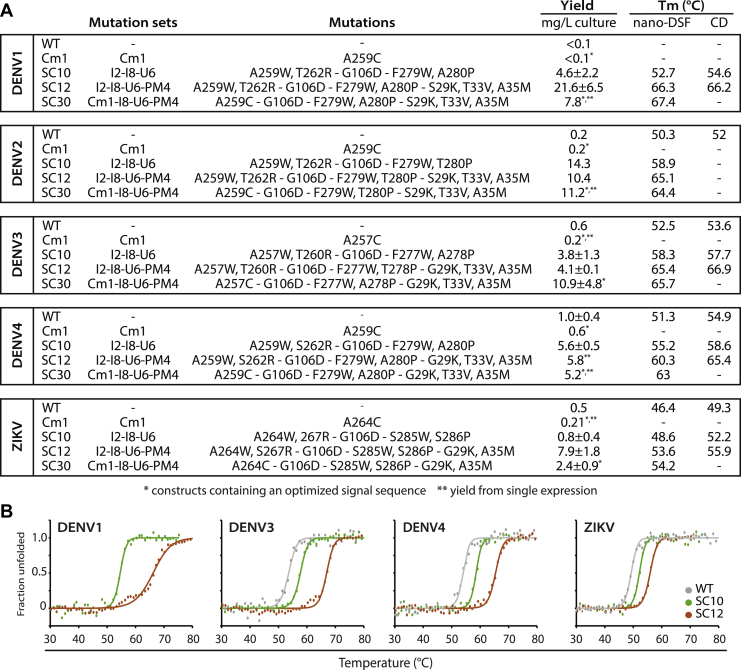
**Rosetta stabilizing combinations (SCs) have increased yield and thermostability compared with WT proteins.***A*, a table of protein yield of SC variants and specific mutations in transient Expi293F expression and *T*_m_ values from nano-DSF and CD. nano-DSF experiments were carried out at 8 μM protein and CD at 10 μM protein. *Dashed values* indicate not determined. Expressions listed are the mean, standard deviation is included if expressed more than three times, and notated if expressed only once. *B*, CD melts for WT (*gray*) at 6.25 μM, SC10 (*green*), and SC12 (*orange*) proteins at 10 μM. WT measurements were obtained from (20) using proteins expressed from CHO DG44 cells. *T*_m_ was interpolated from the fit using Gibbs–Helmholtz equation as described (29). CHO, Chinese hamster ovary; nano-DSF, nano differential scanning fluorimetry.

We selected our best performing stabilizing combinations (SCs) of mutations from our previous work with DENV2 for experimental testing with DENV1/DENV3/DENV4 and ZIKV sE (Figs. 2*A* and S2). The SCs are named based on the conventions we used in our DENV2 sE study (22). For example, DENV2 SC10 and DENV3 SC10 contain the same sets of mutations placed on the DENV2 sE and DENV3 sE background, respectively (specific mutations in Figs. 2 and S2). The Rosetta design variants were expressed in Expi293F cells as secreted proteins and purified using immobilized metal affinity chromatography (Fig. S3*A*).

In our previous study, we showed that the stabilizing mutations in DENV2 sE can lead to large increases in the yield of secreted E protein compared with WT DENV2 sE (up to 70-fold). Here, we similarly found that the mutation sets boost levels of DENV1, 3, 4, and ZIKV sE secreted from cells. The largest enhancement was observed for the DENV1 sE protein variants. DENV1 WT sE secreted at very low levels, <0.1 mg protein per liter of culture, whereas the DENV1 variants had yields ranging from 6.9 mg/l (SC10) to 25.6 mg/l (SC12) (Fig. 2*A*). The SC12 variants were also the highest producing variants for DENV3, DENV4, and ZIKV, boosting production from 4-fold to 250-fold. Larger fold changes in yield were observed for the serotypes in which the WT protein was secreted poorly, particularly DENV1 (Fig. S2).

We also added the stabilizing mutations to covalently linked sE dimers (Cm1), which contain a cysteine mutation (A259C in DENV1/2/4, A257C in DENV3, and A264C in ZIKV) in the central αB helix that forms a disulfide with the symmetric cysteine from the other chain in the homodimer (21). These covalent dimers have similar or lower expression levels compared with WT, even when expressed in stable cell lines (Fig. S2). When the mutations sets I8, U6, and PM4 were added to the Cm1 variants (SC30), expression yields increased by more than eightfold for DENV2, DENV3, and DENV4 (Fig. 2*A*). For DENV1, we were not able to obtain measurable amounts of the Cm1 variant of the WT protein to make a comparison (SC30 expressed at 1.4 mg/l), and for ZIKV, the SC30 variant did not show enhanced yields compared with Cm1.

### DENV and ZIKV design variants are more thermally stable than their WT counterparts

Next, we characterized the thermal unfolding of the DENV and ZIKV design recombinant proteins using nano differential scanning fluorimetry (nano-DSF) and CD. The thermostability of a protein is reflected by their melting temperature at which half of the proteins are unfolded. Thermal transitions can be observed using nano-DSF *via* changes in the intrinsic fluorescence of proteins, whereas with CD, it is the loss of secondary structure within the protein that is measured.

Our nano-DSF measurements at 8 μM sE showed that all DENV and ZIKV SCs had higher *T*_m_ values than their WT counterparts, with increases ranging from 2 to 15 °C (Figs. 2*A* and S3*B*). The I2-I8-U6 mutation set (SC10) led to increases in *T*_m_ of 4 to 8 °C. Protein stability is increased further in all serotypes with the addition of PM4, which was consistent with our findings in DENV2 (22). DENV SCs containing PM4 remain folded up to 60 °C, 10 °C higher than WT proteins (Fig. S2). SC12, which contains I2, I8, U6, and PM4, has the highest thermostability for all four DENV serotypes.

For ZIKV, SC2 (I2-I8-PM4) is the most stable variant, raising the *T*_m_ from 46.4 °C (WT) to 56.5 °C (Fig. S2). Interestingly, the addition of U6 to SC2 lowers the thermostability of the protein to 53.6 °C (SC12, Fig. 2). This may reflect the low sequence conservation between DENV and ZIKV in the hinge region between EDI and EDII. Although the ZIKV SCs are more thermostable than ZIKV WT sE, the *T*_m_ is lower than that of DENV SCs. It is important to note that ZIKV WT sE is 4 to 6 °C less thermally stable than DENVs WT sE, and the SCs did improve *T*_m_ of ZIKV sE by 2 to 8 °C.

CD spectra of the SC proteins at 10 μM (Fig. 2*B*) showed the unfolding of all proteins with increasing temperature. CD results corroborated that the SCs (*brown* and *green*) have higher *T*_m_s than the WT proteins (*gray*). *T*_m_ values extrapolated from CD spectra matched well with our nano-DSF results (Fig. 2*A*). Overall, these results showed that the thermostabilizing mutations from DENV2 also improved sE protein thermostability in DENV1/DENV3/DENV4 and ZIKV.

### Rosetta-stabilizing mutations promote sE dimer formation

**Figure 3:**
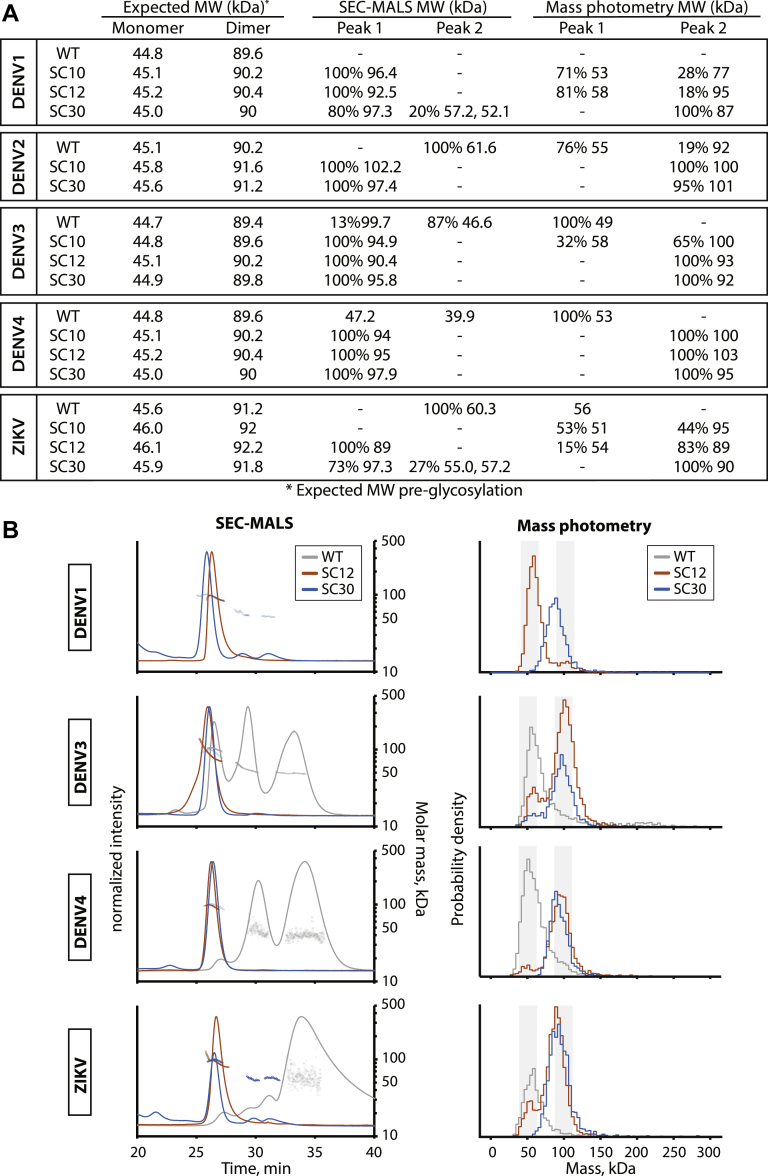
**DENV1/3/4 and ZIKV stabilizing combinations (SCs) have higher homodimer affinity than WT.***A*, comparison between expected molecular weights of sE proteins to MMs of species from SEC–MALS and mass photometry. Relative abundance of each species is represented in percentage. *B*, SEC–MALS chromatograms at 37 °C and mass photometry histograms at room temperature showing distribution of oligomeric states in solution of WT (*gray*), SC12 (*orange*), and SC30 (*blue*) proteins. SEC–MALS experiments were carried out with 100 μl 2.5 mg/ml proteins at 37 °C. WT chromatograms of DENV4 (2.47 mg/ml) and ZIKV (0.97 mg/ml) were obtained (20) using proteins made in CHO DG44 cells. MMs are plotted in *dots*. Mass photometry histograms show *shaded regions* surrounding monomer mass (∼50 kDa) and dimer mass (∼100 kDa). CHO, Chinese hamster ovary; DENV, dengue virus; MM, molar mass; sE, soluble E protein; SEC–MALS, size-exclusion chromatography coupled with multiangle light scattering experiment; ZIKV, Zika virus.

To assess dimer formation at physiological temperature, we performed size-exclusion chromatography coupled with multiangle light scattering experiment (SEC-MALS) at 37 °C. We previously established that the WT proteins for DENV and ZIKV are weak homodimers and are primarily monomeric at 37 °C (20) (Fig. 3). DENV3 WT migrates as three species by SEC. Approximately 13% of the protein eluted with a measured molar mass (MM) from MALS of 100 kDa, consistent with formation of a homodimer. For the remaining protein, which eluted as two species, the light scattering indicates both peaks were monomers (MM near 47 kDa), suggesting that the DENV3 monomer exists in two different monomer conformations. Similarly, DENV4 WT sE eluted in two peaks, both with measured MMs close to that of a monomer. ZIKV WT sE eluted in one peak with a fitted molecular weight of 60.3 kDa, which is between the expected weights for a monomer and dimer. We were unable to purify enough WT DENV1 sE to perform SEC–MALS. These results show that WT sE proteins exist in an equilibrium between monomer and dimer that favors the monomeric state at 37 °C.

SEC–MALS experiments were performed with the Rosetta E protein variants under the same conditions to study the effect of the stabilizing mutations on homodimer affinity. We found that for all serotypes, SC10 and SC12 eluted in a single peak with a measured MM close to the expected mass for a homodimer (Fig. 3, *A* and *B*). As expected, the stabilized covalent dimer, SC30, was dimeric *via* SEC–MALS for all the proteins. We observed that I2 played an important role in stabilizing the sE homodimer for DENV1, DENV3, DENV4, and ZIKV (Fig. S2). SC29, which lacks I2, migrates either as a mixture between monomers and dimers (DENV3 and ZIKV) or as entirely monomer (DENV1).

One disadvantage of SEC–MALS is that the protein concentrations needed to make a measurement (∼5 μM for sE) are higher than the typical nanomolar concentrations used during vaccination. To assess dimer stability at lower protein concentrations, we performed mass photometry, a single-molecule technique, which uses light scattering events to infer masses in solution (23). We collected mass photometry measurements on 50 nM sE at room temperature to assess the oligomeric state of the protein at lower protein concentrations. All the WT sE proteins (DENV1 and 3–4 as well as ZIKV) presented as a single species with molecular weights near the expected mass of a monomer, ∼50 kDa (Fig. 3). In contrast, the design variants displayed a range of behaviors with some variants mostly dimeric and some predominately in the monomeric state. Aside from the covalent dimer (SC30), which presented as 100% dimer, the SC12 variants displayed the highest preference for the dimeric state with DENV4 SC12, DENV3 SC12, and ZIKV SC12, all greater than 80% dimer at a concentration of 50 nM. In contrast, DENV1 SC12 was ∼80% monomer and ∼20% dimer under these conditions. Interestingly, ZIKV SC29 is completely dimeric at conditions used for mass photometry yet migrated as a single species with MM between monomer and dimer on SEC–MALS at 37 °C (Fig. S2), indicating that this equilibrium is highly sensitive to changes in temperature. Consistent with our previously published results, DENV2 SC10 was 100% dimer at 50 nM (Fig. S4, (22)). These results confirm that the Rosetta mutations stabilize the dimeric form of all DENV serotypes and ZIKV at micromolar concentrations at 37 ˚C and at low nanomolar concentrations, with the exception of DENV1 sE remaining primarily a monomer in solution at lower concentrations.

### The stabilized sE proteins display monomer and dimer epitopes at physiological temperature

**Figure 4:**
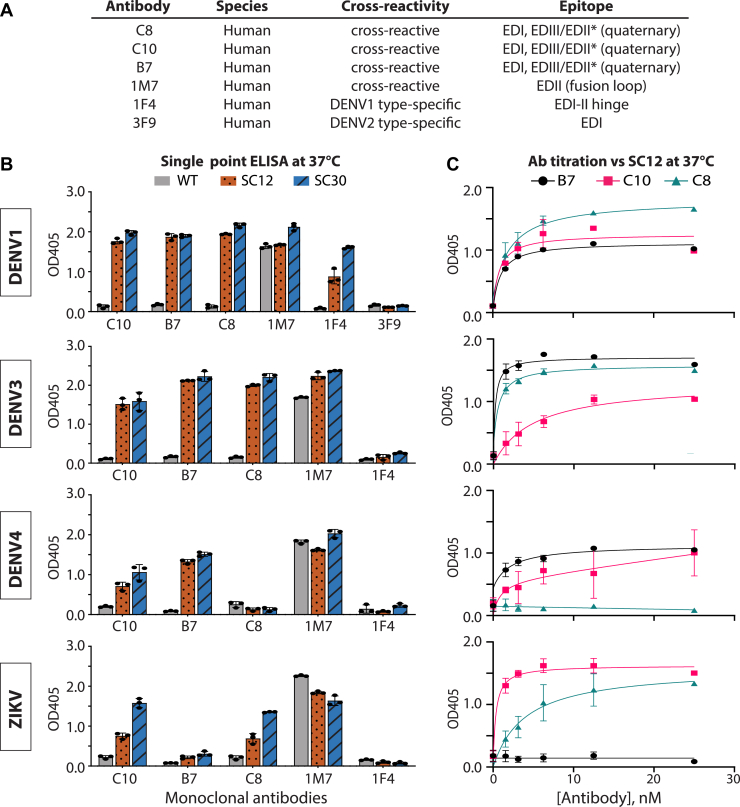
**DENV1/3/4 and ZIKV Rosetta stabilizing combinations (SCs) display E monomer and dimer epitopes at physiological temperature.***A*, tested human monoclonal antibodies and their corresponding epitope on the E ectodomain. A panel of antibodies binding to type-specific or cross-reactive epitopes was used to assess the epitope presentation of sE variants. ∗ indicates domains on a different monomer. *B*, binding of monoclonal antibodies to WT (*gray*), SC12 (*orange*, *dotted*), and SC30 (*blue*, *striped*) in single point ELISA at 37 °C. About 45 nM sE was immobilized on nickel ELISA plate and incubated with 2 ng/μl (∼13 nM) monoclonal IgG. 3F9 was used as a negative control for DENV1 experiments and 1F4 as a negative control for DENV3/4 and ZIKV. Individual data points represent technical triplicates; bars show mean ± standard deviation. *C*, titration ELISA showing binding of SC12 proteins to quaternary epitope antibodies B7 (*black circle*), C10 (*pink square*), and C8 (*teal triangle*) at increasing antibody concentration (duplicate mean ± standard error). DENV, dengue virus; IgG, immunoglobulin G; sE, soluble E protein; ZIKV, Zika virus.

We next examined if the Rosetta design variants bind to mAbs known to neutralize DENV and/or ZIKV. We used a panel of serotype-specific and crossreactive mAbs (10, 13, 14, 17, 24, 25, 26) that bind to well-defined and previously characterized epitopes on DENV and ZIKV E protein (Fig. 4*A*). In particular, we focused on the EDE mAbs C8, C10, and B7 (14). These mAbs are strongly neutralizing toward DENVs produced by either insect (C6/36) or human monocyte-derived dendritic cells with 50% neutralization titer at subnanomolar concentrations. In addition, they are also equally neutralizing toward ZIKV (17).

We performed ELISAs at 37 °C by immobilizing the His-tagged sE proteins on nickel plates followed by incubation with the mAbs. sE proteins were prepared at a concentration of 45 nM prior to immobilization. In previous studies, we demonstrated that this traps the sE protein in its prevalent oligomeric state and allows for indirect assessment of sE dimer stability (22). Consistent with the SEC–MALS experiments, at 37 °C, the WT sE proteins did not bind to EDE mAbs, whereas the most stable Rosetta variants, SC12 and SC30, displayed strong equivalent binding to all quaternary/dimer epitope mAbs tested (Fig. 4*B*). Surprisingly, the DENV4 proteins showed low binding to the EDE mAb C8 but bound strongly to C10 and B7.

The difference between class 1 and 2 of EDE (EDE1 or EDE2) antibodies is a sensitivity to glycosylation at N153 (N154 in ZIKV) (14, 17). We found that ZIKV sE proteins bound well to EDE1 mAbs C8 and C10 but poorly to EDE2 mAb B7, consistent with previously reported structural studies of ZIKV WT sE and EDE mAb complexes (17). This is due to ZIKV sE having a different conformation in the 150 loop that changes the position of the N154 glycan, which is an important contact for EDE2 mAbs. These results indicate that Rosetta design variants were able to present the crossdimer epitope necessary for EDE antibody (Ab) binding.

For monomer epitope mAbs, we found that 1M7 had high binding to all antigens tested, including WT proteins (Fig. 4*B*). 1M7 is a cross-reactive Ab that binds to the fusion loop epitope present within each DENV and ZIKV E protein and is conserved across flaviviruses (26). For the DENV1 type–specific Ab 1F4, the stable dimers bound better to the mAb compared with the WT sE protein, even though the epitope has previously mapped to a monomer. In addition, 1F4 binds more tightly to the cysteine dimer (SC30) of DENV1 sE than it does SC12. The 1F4 epitope is predominately in EDI of the E protein but extends into the EDI/II hinge region, and binding is sensitive to the angle of approach of the immunoglobulin G (IgG) to the ectodomain and the conformation of the EDI/II hinge (25). At nanomolar concentrations, DENV1 SC12 is in equilibrium between a monomer and dimer, whereas SC30 is a covalent dimer. Dimerization may stabilize the EDI/II hinge in a conformation that favors binding to 1F4. However, we did observe full binding between DENV1 SC12 and the antidimer antibodies C8, C10, and B7.

In addition, we found that DENV3 SC variants presented epitopes on EDI that are required for interactions with previously characterized DENV3 type–specific mAbs (Fig. S5). Importantly, DENV3 SC12 and SC30 bind better to these antibodies than WT, which is consistent with reported results using DENV3 sE Cm1 (27).

We also used ELISA experiments to monitor binding as a function of mAb concentration between intradimer mAbs and the SC12 variants (Fig. 4*C*). About 45 nM sE was incubated with varying concentrations of mAb at 37 °C. Our results show that the binding was saturated at 12.5 nM mAb. DENV1 SC12 bound equally well to all dimer mAbs, whereas DENV3 SC12 bound weaker to C10. Weaker binding of DENV3 SC12 to C10 is consistent with neutralization experiments that have shown that B7 and C8 neutralize DENV3 at lower Ab concentrations than C10 (14, 17). As reflected in the single-point ELISA data (Fig. 4*B*), DENV4 SC12 showed minimal binding to C8, and ZIKV SC12 did not bind to B7.

## Discussion

We identified mutations that increase the expression and protein stability of sE from all four serotypes of DENV and ZIKV. These mutations were first identified during re-engineering of DENV2 sE using the protein design software Rosetta (22) and are located in conserved regions of the DENV and ZIKV E ectodomain (Fig. S1). The mutation sets I2, I8, and U6 primarily stabilize dimer formation, whereas the mutation set PM4 also raises the temperature at which the sE protein monomer unfolds. PM4 contains three mutations, all located in the core of EDI. The fact that PM4 raises the *T*_m_ of all five proteins suggests that the low intrinsic stability of WT EDI is conserved between the four serotypes and ZIKV. The amount of secreted protein (*i.e.*, expression yield) was sensitive to both dimer stability and the thermal unfolding temperature of sE. Dimer formation sequesters the fusion loop at the interface between the two chains of the homodimer and likely disfavors insertion of the fusion loop into membranes of the host cell, thus allowing for better protein secretion.

The effects of the mutations on dimer stability were not equal across all DENV serotypes and ZIKV. The DENV1, DENV3, and DENV4 sE proteins share 63 to 67% sequence identity to DENV2 sE, and ZIKV sE is 54% identical to DENV2. Because the amino acids surrounding each mutation site are highly conserved among these proteins (Fig. S1), it is likely that amino acids outside these regions also play an important role in determining dimer stability. Further mutations in these regions may be needed to create an obligate homodimer at low protein concentrations for DENV1 sE and ZIKV sE.

The binding experiments with mAbs that recognize quaternary epitopes on the virus indicate that the stabilized dimers are adopting conformations that resemble the conformation of the E protein on the surface of the virus. We did observe an unexpected result with our sE variants from DENV4 in that weaker binding was observed to the mAb C8. Swanstrom *et al.* (16) reported that C8 and C10 bind weaker to DENV4 than other viruses but to similar levels with each other. Previously, C8 was reported to neutralize DENV4 as effectively as other DENVs (14, 17). Our results suggest that the C8 epitope may be presented differently on the virus than it is on the stabilized DENV4 sE variants, but other EDE epitopes (such as C10 and B7) are still maintained.

In considering the development of subunit vaccines for DENV and ZIKV, high protein expression and stability is important for industrial production of purified protein and may also be important for mRNA-based vaccines that rely on secretion (or display) of the protein from a patient’s cells. These mutations bring major advantages, especially for DENV1, as they allow for more protein to be produced in a small-scale transient expression compared with requiring a large-scale production with use of a stable cell line. As we learn more about Ab responses toward flaviviruses, it is clear that antibodies capable of recognizing quaternary epitopes are potently neutralizing (15, 28). Among these are antibodies that bind across the E homodimer, such as type-specific mAbs 2D22 (DENV2) or G9E (ZIKV) and EDE class antibodies. Thus, the ability to stabilize sE to present quaternary epitopes has great potential and can lead to a new class of second-generation vaccines. Our work has demonstrated that the Rosetta-stabilizing mutations are great tools to generate new subunit vaccines for DENV and ZIKV, as they significantly enhanced recombinant E homodimer stability and allow for proper display of important quaternary Ab epitopes.

A critical lesson from the clinical use of Dengvaxia is that a safe DENV vaccine should elicit a potent response against all four serotypes. One potential advantage of a tetravalent subunit vaccine is that there may be more precise control over the response against each serotype as the amount of each antigen can be explicitly controlled in product formulation. In future studies, it will be important to examine if the stabilized sE variants elicit an even response if formulated as a tetravalent vaccine or if a subset of the sE proteins are immunodominant.

## Experimental procedures

### Plasmids and genes

DENV1/3/4 and ZIKV sE consisted of the ectodomain of the E protein of each virus. Amino acid sequences were chosen based on the World Health Organization reference strains for DENV1 (WestPac; catalog no.: AAN06982; amino acids 1–395), DENV3 (catalog np.: CH53489; amino acids 1–395), DENV4 (catalog no.: TVP-376; amino acids 1–397), and ZIKV (catalog no.: H/PF/2013; amino acids 1–404). Rosetta mutations were introduced to DENV1/3/4 and ZIKV sE at the same positions as in DENV2 based on multiple sequence alignment. A human serum albumin signal sequence was included at the N terminus of sE, and a C-terminal 8× His tag was added for all Rosetta variants (6× His tag for WT). The coding sequences were codon optimized for mammalian systems and cloned into the mammalian expression vector PaH. Expression was driven by a CAG promoter.

### Expression and purification of DENV1, DENV3, DENV4, and ZIKV sE

sE proteins were expressed in Expi293F mammalian cells by transient transfection (Thermo Fisher Scientific; catalog nos.: A14527 and A14525) using the manufacturer-supplied protocols, unless otherwise indicated. Cultures were harvested 24 h postenhancement, and the media were clarified by centrifugation and filtration through a 0.22 μm membrane. sE proteins containing an 8× His tag were purified from supernatant using immobilized metal affinity chromatography with Ni–Penta agarose 6FF (PROTEINDEX; catalog no.: 11-0227-010). Supernatants were concentrated with Amicon filters and allowed to bind to Ni–Penta agarose for 1 h at 4 °C. After the supernatant was flowed through a column, the resin was washed with 50 mM Tris–HCl, 1 M NaCl, and 25 mM imidazole at pH 8.0. The resin was further washed with 1× PBS (137 mM NaCl, 2.7 mM KCl, 8 mM Na_2_HPO_4_, and 2 mM KH_2_PO_4_) containing 25 mM imidazole and then with 1× PBS containing 50 mM imidazole at pH 7.4. Proteins were serially eluted with 1× PBS (pH 7.4) containing 500 mM imidazole. Following elution, samples were concentrated and buffer exchanged using Zeba Spin Desalting Columns, 7 K molecular weight cutoff, 0.5 ml to 1× PBS. Samples were stored in 1× PBS at 4 °C.

### Nano-DSF of DENV1/3/4 and ZIKV sE proteins

Nano-DSF thermal unfolding experiments were performed on a Prometheus NT.48 system (NanoTemper). Proteins were diluted to 8 μM sE in 1× PBS (pH 7.4), and 10 μl of each protein was loaded into a capillary. The protein samples were subjected to a heating regime of 1 °C/min from 20 to 95 °C. The intrinsic fluorescence signals at 330 and 350 nm were measured for each experiment. The *T*_m_ was calculated automatically based on the first derivative of fluorescence signal by the software PR.ThermControl (NanoTemper). The *T*_m_ values reported in this study were extrapolated from the first derivative of 350 nm fluorescence signal.

### CD of DENV1/3/4 and ZIKV sE proteins

CD spectra of 10 μM protein were collected using a Jasco J-815 CD spectrometer. Samples were diluted in 1× PBS (pH 7.4), and 200 μl was added to a 1-mm glass quartz cuvette. Thermal unfolding was measured at 213 nm from 20 to 95 °C at a ramping rate of 2 °C/min. The mean residue ellipticity for each protein was calculated using n values of 394, 392, 394, and 404 for DENV1, DENV3, DENV4, and ZIKV sE, respectively, and fit to the Gibbs–Helmholtz equation described (29) to determine the *T*_m_.

### SEC–MALS at 37 °C

SEC–MALS experiments were performed using a GE Superdex 200 10/300 GL column–equipped Agilent FPLC system interfaced with a Wyatt DAWN HELEOS II light-scattering instrument, Wyatt T-rEX refractometer, and a Wyatt dynamic light scattering module. The column was pre-equilibrated in 0.2 μm filtered running buffer of 1× PBS + 0.02% w/v sodium azide before each injection. To equilibrate the system at 37 °C, the running buffer was incubated in a water bath and the column was wrapped with THG thermal tape with a digital thermostat regulator (22). For DENV1/3/4 and ZIKV Rosetta design variants, 100 μl of 2.5 mg/ml ice-cold protein (250 μg) was injected to the column at a flow rate of 0.5 ml/min in 1× PBS + 0.02% w/v sodium azide. WT proteins produced from stable Chinese hamster ovary DG44 cells were used for SEC–MALS and were injected in varying amounts as previously published (20): DENV3 at 2.5 mg/ml, DENV4 at 2.47 mg/ml, and ZIKV at 0.97 mg/ml. MALS data were collected and analyzed using Wyatt ASTRA software, version 11.

### Mass photometry

Mass photometry experiments were done using Refeyn System according to manufacturer’s instructions. Prior to each measurement, glass slides (Thorlabs; catalog no.: 0107222) were cleaned by bath sonication two times in 50% isopropanol (10 min each) and a final sonication step in ultrapure water. Cleaned glass slides were stored in ultrapure water until used. To prepare for each measurement, a clean glass slide was dried using gentle air flow. A silicone gasket (Grace Biolabs; catalog no.: 103250) was adhered to the slide. The slide was placed on top of the laser beam (gasket side up) with a thin layer of immersion oil in between. Data were acquired using the AcquireMP software (Refeyn). For each measurement, the instrument performed a coarse focus mode (buffer free) on the gasket well and then 15 to 20 μl 50 nM sE was carefully dropped into the well to continue fine focusing and recording of events on the glass slide. Protein species in the sample were evaluated by the DiscoverMP software (Refeyn) based on the contrast values measured from their light scattering property. All mass photometry measurements were performed at room temperature.

### ELISA against mAbs

ELISA experiments were carried out on nickel-coated ELISA plates (Pierce; catalog no.: 15442). All incubation steps were done at 37 °C with 300 rpm shaking. To bind antigens to the plate, 50 μl 45 nM sE protein was added to each well and allowed to incubate for 1 h. Wells were then washed with 200 μl 1× 20 mM Tris (pH 7.6), 150 mM NaCl, and 0.2% Tween-20 three times *via* aspiration and blocked with 1× Tris-buffered saline containing 3% w/v nonfat dry milk and 0.05% Tween-20 for another hour. Wells were washed three times with washing buffer postblocking. About 50 μl primary Ab (human monoclonal C8, C10, B7, 1M7, 1F4, and 3F9, and DV3 type–specific mAbs) at 2 ng/μl in blocking buffer was added to each well and allowed to bind to the antigen for 1 h. After three washing steps, 50 μl alkaline phosphatase–conjugated goat antihuman IgG secondary Ab at 1:10,000 dilution was added to the plate for 45 min. After three final washing steps, the signal was developed using the alkaline phosphatase substrate *para*-nitrophenyl phosphate (Southern Biotech; catalog no.: 0421-01L) (50 μl/well). Binding responses were reported as the absorbance at 405 nm. Error bars are standard deviation from technical triplicates or standard error from duplicates as indicated in the figure legend.

## Data availability

All data described in this article are contained within the article and the supporting information.

## Supporting information

This article contains supporting information (20).

## Conflict of interest

The authors declare that they have no conflicts of interest with the contents of this article.
