# Supplementary material for: A conserved set of mutations for stabilizing soluble envelope protein dimers from dengue and Zika viruses to advance the development of subunit vaccines
Source: J Biol Chem. 2022 May 26;298(7):102079. doi: 10.1016/j.jbc.2022.102079 (PMC9249817; doi:10.1016/j.jbc.2022.102079)
Supplement: Supplemental Figures S1–S5 [file mmc1.pdf]

A conserved set of mutations for stabilizing soluble envelope protein dimers from Dengue and Zika viruses to advance the development of subunit vaccines

Thanh T. N. Phan[1*], Matthew G. Hvasta[1*], Stephan T. Kudlacek[1], Devina J. Thiono[2], Ashutosh Tripathy[1], Nathan I. Nicely[3], Aravinda M. de Silva[2], Brian Kuhlman[1,+]

From the [1]Department of Biochemistry and Biophysics, University of North Carolina at Chapel Hill, NC 27599, USA, the[2]Department of Microbiology and Immunology, University of North Carolina at Chapel Hill, NC 27599, USA, and the [3]Department of Pharmacology, University of North Carolina at Chapel Hill, NC 27599.

*These authors contributed equally

Running Title: Stabilization of DENVs and ZIKV soluble E

[+]To whom correspondence should be addressed: Prof. Brian Kuhlman, Department of Biochemistry and Biophysics, University of North Carolina at Chapel Hill, NC 27599, USA; email: bkuhlman@email.unc.edu

Current address: Stephan Kudlacek, Menten AI

Keywords: antigen presentation, protein design, Rosetta, protein stability, vaccine development, Dengue virus, Zika virus, flavirirus, soluble E dimer, antibody binding

Table of Contents **Page**

Figure S1: Multiple sequence alignment of DENV1-4 and ZIKV sE S-2

Figure S2: Table with all protein mutations, expression, and characterization results S-3

Figure S3: SDS-PAGE and nano-DSF comparing WT to SC10,12, 30 S-4

Figure S4: Mass Photometry of DENV2 SC10 S-5

Figure S5: Single point ELISA of DV3 SC proteins against DV3 type-specific mAbs at 37°C S-6

Supporting Information

```
                 mutated positions   contacts   P4   I8   I2   U6

DENV2   MRCIGISNRDFVEGVSGGSWVDIVLEHGSCVTTMAKNKPTLDFELIKTEAKQPATLRKYC    60
DENV1   MRCVGIGNRDFVEGLSGATWVDVVLEHGSCVTTMAKDKPTLDIELLKTEVTNPAVLRKLC    60
DENV3   MRCVGVGNRDFVEGLSGATWVDVVLEHGGCVTTMAKNKPTLDIELQKTEATQLATLRKLC    60
DENV4   MRCVGVGNRDFVEGVSGGAWVDLVLEHGGCVTTMAQGKPTLDFELTKTTAKEVALLRTYC    60
ZIKV    IRCIGVSNRDFVEGMSGGTWVDIVLEHGGCVTVMAQDKPTVDIELVTTTVSNMAEVRSYC    60
        :**:*:.*******:**.:***:*****.***.**:.***:*:** .* ..: * :*. *

DENV2   IEAKLTNTTTESRCPTQGEPSLNEEQDKRFVCKHSMVDRGWGNGCGLFGKGGIVTCAMFT    120
DENV1   IEAKISNTTTDSRCPTQGEATLVEEQDTNFVCRRTFVDRGWGNGCGLFGKGSLITCAKFK    120
DENV3   IEGKITNITTDSRCPTQGEAILPEEQDQNYVCKHTYVDRGWGNGCGLFGKGSLVTCAKFQ    120
DENV4   IEASISNITTATRCPTQGEPYLKEEQDQQYICRRDVVDRGWGNGCGLFGKGGVVTCAKFS    120
ZIKV    YEASISDMASDSRCPTQGEAYLDKQSDTQYVCKRTLVDRGWGNGCGLFGKGSLVTCAKFA    120
         *..::: :: :*******  * ::.* .::*::  ***************.::*** *

DENV2   CKKNMEGKVVQPENLEYTIVVTPHSGEEH-----AVGNDTGKHGKEIKVTPQSSITEAEL    175
DENV1   CVTKLEGKIVQYENLKYSVIVTVHTGDQH-----QVGNETTEHGTTATITPQAPTSEIQL    175
DENV3   CLESIEGKVVQHENLKYTVIITVHTGDQH-----QVGNET--QGVTAEITPQASTVEAIL    173
DENV4   CSGKITGNLVQIENLEYTVVVTVHNGDTH-----AVGNDTSNHGVTAMITPRSPSVEVKL    175
ZIKV    CSKKMTGKSIQPENLEYRIMLSVHGSQHSGMIVNDTGHETDENRAKVEITPNSPRAEATL    180
        *  .: *: :* ***:* :::: * .:        .*::*  :     :**.:   *  *

DENV2   TGYGTVTMECSPRTGLDFNEMVLLQMENKAWLVHRQWFLDLPLPWLPGADTQGSNWIQKE    235
DENV1   TDYGALTLDCSPRTGLDFNEMVLLTMEKKSWLVHKQWFLDLPLPWTSGASTSQETWNRQD    235
DENV3   PEYGTLGLECSPRTGLDFNEMILLTMKNKAWMVHRQWFFDLPLPWTSGATTETPTWNRKE    233
DENV4   PDYGELTLDCEPRSGIDFNEMILMKMKKKTWLVHKQWFLDLPLPWTAGADTSEVHWNYKE    235
ZIKV    GGFGSLGLDCEPRTGLDFSDLYYLTMNNKHWLVHKEWFHDIPLPWHAGADTGTPHWNNKE    240
          :* : ::*.**:*:**.::  : *::* *:**::** *:****  ** *    *  ::

DENV2   TLVTFKNPHAKKQDVVVLGSQEGAMHTALTGATEIQMSSG-NLLFTGHLKCRLRMDKLQL    294
DENV1   LLVTFKTAHAKKQEVVVLGSQEGAMHTALTGATEIQTSGT-TTIFAGHLKCRLKMDKLTL    294
DENV3   LLVTFKNAHAKKQEVVVLGSQEGAMHTALTGATEIQNSGG-TSIFAGHLKCRLKMDKLEL    292
DENV4   RMVTFKVPHAKRQDVTVLGSQEGAMHSALAGATEVDSGDG-NHMFAGHLKCKVRMEKLRI    294
ZIKV    ALVEFKDAHAKRQTVVVLGSQEGAVHTALAGALEAEMDGAKGRLSSGHLKCRLKMDKLRL    300
         :* **  ***:* *.********:*:**:** * : ..    : :*****:::*:** :

DENV2   KGMSYSMCTGKFKVVKEIAETQHGTIVIRVQYEGDGSPCKIPFEI-MDLEKRHVLGRLIT    353
DENV1   KGMSYVMCTGSFKLEKEVAETQHGTVLVQVKYEGTDAPCKIPFSS-QDEKGVTQNGRLIT    353
DENV3   KGMSYAMCLNTFVLKKEVSETQHGTILIKVEYKGEDAPCKIPFST-EDGQGKAHNGRLIT    351
DENV4   KGMSYTMCSGKFSIDKEMAETQHGTTVVKVKYEGAGAPCKVPIEI-RDVNKEKVVGRIIS    353
ZIKV    KGVSYSLCTAAFTFTKIPAETLHGTVTVEVQYAGTDGPCKVPAQMAVDMQTLTPVGRLIT    360
        **:** :*   * . *  :** ***  :.*:* * ..***:* .   * :     **:*:

DENV2   VNPIVTEKD--SPVNIEAEPPFGDSYIIIGVDPGQLKLNWFKK-    394
DENV1   ANPIVTDKE--KPVNIEAEPPFGESYIVVGAGEKALKLSWFKK-    394
DENV3   ANPVVTKKE--EPVNIEAEPPFGESNIVIGIGDKALKINWYKK-    392
DENV4   STPLAENTN--SVTNIELEPPFGDSYIVIGVGNSALTLHWFRK-    394
ZIKV    ANPVITESTENSKMMLELDPPFGDSYIVIGVGEKKITHHWHRSG    404
        .*:  ..   .   :* :****:* *::* .   :.  *.:.
```

Figure S1. Multiple sequence alignment of DENV1-4 and ZIKV sE sequences. Positions corresponding to mutation sets PM4 (red), I2 (green), I8 (blue) and U6 (yellow) are highlighted in solid colors with white text. Sidechain contacts are lightly highlighted with black text. Contacts were determined by visual inspection using the crystal structure for DENV2 sE SC10 (PDB 6WY1) and the Rosetta model for DENV2 sE with PM4 mutations.

				Yield	Tm (°C)		Expected Molar Mass*** (kDa)		SEC-MALS MW (kDa)		Mass photometry MW (kDa)	
		Mutation set	Mutations	mg/L culture	nano-DSF 8 ∝M @ 350 nm	CD 10 ∝M @ 213 nm	Monomer	Dimer	Peak 1	Peak 2	Peak 1	Peak 2
DENV1	WT	-	-	<0.1	-	-	44.8	89.6	-	-	-	-
	Cm1	-	A259C	<0.1*,**	-	-	44.8	89.6	-	-	-	-
	SC2	I2-I8-PM4	A259W, T262R - G106D - S29K, T33V, A35M	6.2**	64.4	66	45.1	90.2	-	-	-	-
	SC10	I2-I8-U6	A259W, T262R - G106D - F279W, A280P	4.6±2.2	52.7	54.6	45.1	90.2	100% 96.4	-	71% 53	28% 77
	SC12	I2-I8-U6-PM4	A259W, T262R - G106D - F279W, A280P - S29K, T33V, A35M	21.6±6.5	66.3	66.2	45.2	90.4	100% 92.5	-	81% 58	18% 95
	SC14	I2-U6	A259W, T262R - F279W, A280P	1.0±0.3	50.8	-	45	90	-	-	-	-
	SC29	I8-U6-PM4	G106D - F279W, A280P - S29K, T33V, A35M	3.1±1.0	66	67.5	45.0	90	-	100% 53.6	92% 51	-
	SC30	Cm1-I8-U6-PM4	A259C - G106D - F279W, A280P - S29K, T33V, A35M	7.8*,**	67.4	-	45.0	90	80% 97.3	20% 57.2, 52.1	-	100% 87
DENV2	WT	-	-	0.2	50.3	52	45.1	90.2	-	100% 61.6	76% 55	19% 92
	Cm1	-	A259C	0.2*	-	-	45.1	90.2	100% 100.7	-	12% 60	84% 104
	SC10	I2-I8-U6	A259W, T262R - G106D - F279W, T280P	14.3	58.9	-	45.8	91.6	100% 102.2	-	-	100% 100
	SC12	I2-I8-U6-PM4	A259W, T262R - G106D - F279W, T280P - S29K, T33V, A35M	10.4	65.1	-	45.7	91.4	-	-	-	92% 101
	SC14	I2-U6	A259W, T262R - F279W, T280P	11.4	57.8	-	45.6	91.2	100% 99.5	-	5% 56	88% 91
	SC30	Cm1-I8-U6-PM4	A259C - G106D - F279W, T280P - S29K, T33V, A35M	11.2*	64.4	-	45.6	91.2	100% 97.4	-	-	95% 101
DENV3	WT	-	-	0.6	52.5	53.6	44.7	89.4	13% 99.7	87% 46.6	100% 49	-
	Cm1	-	A257C	0.2*	52.5	53.6	44.7	89.4	-	-	-	-
	SC2	I2-I8-PM4	A257W, T260R - G106D - G29K, T33V, A35M	1.6**	-	-	45	90	-	-	-	-
	SC10	I2-I8-U6	A257W, T260R - G106D - F277W, A278P	3.8±1.3	58.3	57.7	44.8	89.6	100% 94.9	-	32% 58	65% 100
	SC12	I2-I8-U6-PM4	A257W, T260R - G106D - F277W, A278P - G29K, T33V, A35M	4.1±0.1	65.4	66.9	45.1	90.2	100% 90.4	-	-	100% 93
	SC14	I2-U6	A257W, T260R - F277W, A278P	2.8±0.5	55.5		44.9	89.8	96.4% 93.6	3.6% 64.6	61% 63	31% 100
	SC29	I8-U6-PM4	G106D - F277W, A278P - G29K, T33V, A35M	4.5±0.1	64.5	65.9	44.9	89.8	3% 96	77% 82, 20% 55	47% 53	63% 85
	SC30	Cm1-I8-U6-PM4	A257C - G106D - F277W, A278P - G29K, T33V, A35M	10.9±4.8*	65.7	-	44.9	89.8	100% 95.8	-	-	100% 92
DENV4	WT	-	-	1.0±0.4	51.3	54.9	44.8	89.6	47.2	39.9	100% 53	-
	Cm1	-	A259C	0.6*,**	-	-	45.0	90.0	-	-	-	-
	SC2	I2-I8-PM4	A259W, S262R - G106D - G29K, T33V, A35M	4.2±2.1	64.1	63.8	45.1	90.2	-	-	-	
	SC10	I2-I8-U6	A259W, S262R - G106D - F279W, A280P	5.6±0.5	55.2	58.6	45.1	90.2	100% 94	-	-	100% 100
	SC12	I2-I8-U6-PM4	A259W, S262R - G106D - F279W, A280P - G29K, T33V, A35M	5.8**	60.3	65.4	45.2	90.4	100% 95	-	-	100% 103
	SC29	I8-U6-PM4	G106D - F279W, A280P - G29K, T33V, A35M	6.2±0.82	60.6	64.1	45.0	90	100% 83.0	-	47% 45	47% 87
	SC30	Cm1-I8-U6-PM4	A259C - F279W, A280P - G29K, T33V, A35M	5.2*,**	63	-	45.0	90	100% 97.9	-	-	100% 95
ZIKV	WT	-	-	0.5**	46.4	49.3	45.6	91.2	-	100% 60.3	56	-
	Cm1	-	A264C	0.21*,**	-	-	45.6	91.2	-	-	-	-
	SC2	I2-I8-PM4	A264W, S267R - G106D - G29K, A35M	8.8**	56.5	58.2	46.0	92.0	-	-	-	-
	SC10	I2-I8-U6	A264W, S267R - G106D - S285W, S286P	0.8±0.4	48.6	52.2	46.0	92	-	-	53% 51	44% 95
	SC12	I2-I8-U6-PM4	A264W, S267R - G106D - S285W, S286P - G29K, A35M	7.9±1.8	53.6	55.9	46.1	92.2	100% 89	-	15% 54	83% 89
	SC29	I8-U6-PM4	G106D - S285W, S286P - G29K, A35M	3.1±1.9	52.6	53.4	45.9	91.8	100% 70.6	-	-	100% 94
	SC30	Cm1-I8-U6-PM4	A264C - G106D - S285W, S286P - G29K, A35M	2.4±.9*	54.2	-	45.9	91.8	73% 97.3	27% 55.0, 57.2	-	100% 90

* constructs containing an optimized signal sequence, ** yield from single expression, *** Expected MW pre-glycosylation

Figure S2. Master table of biophysical characterization for the stabilized combinations. (-) indicates data not obtained. Expressions listed are the mean, standard deviation is included if expressed more than three times, and notated if expressed only once. CD and SEC-MALS measurements for WT and Cm1 proteins were published from Kudlacek et al. (2018).

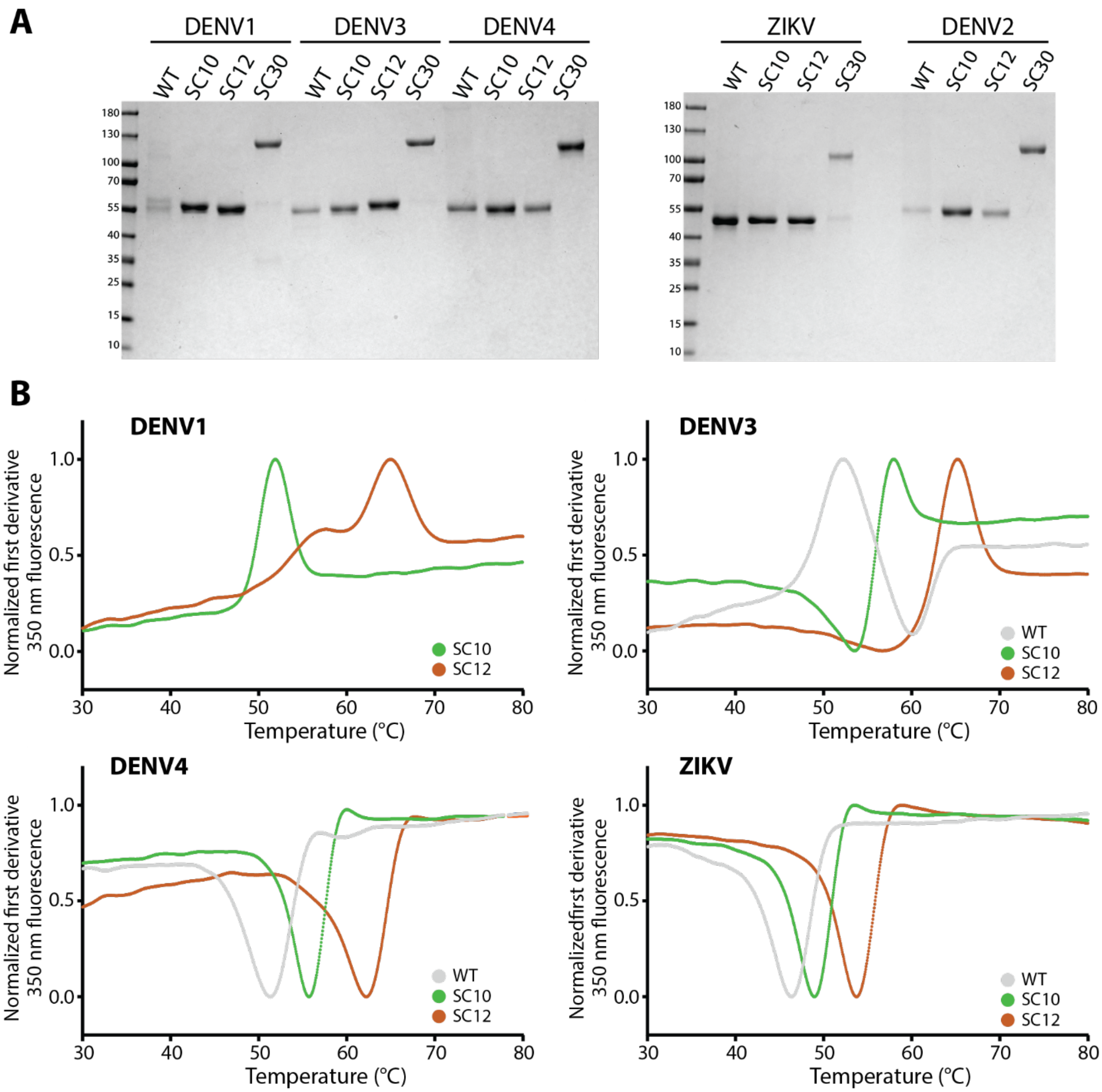

Figure S3. Expression and thermostability of DENV1/3/4 and ZIKV SCs. (A) Non-reducing SDS-PAGE showing purified proteins from immobilized metal affinity purification (1 µg protein). (B) Nano-DSF data showing the thermal unfolding of 8 µM WT (grey), SC10 (green) and SC12 (orange). T_m values were determined by the positive peak of the derivative of fluorescence signal at 350 nm.

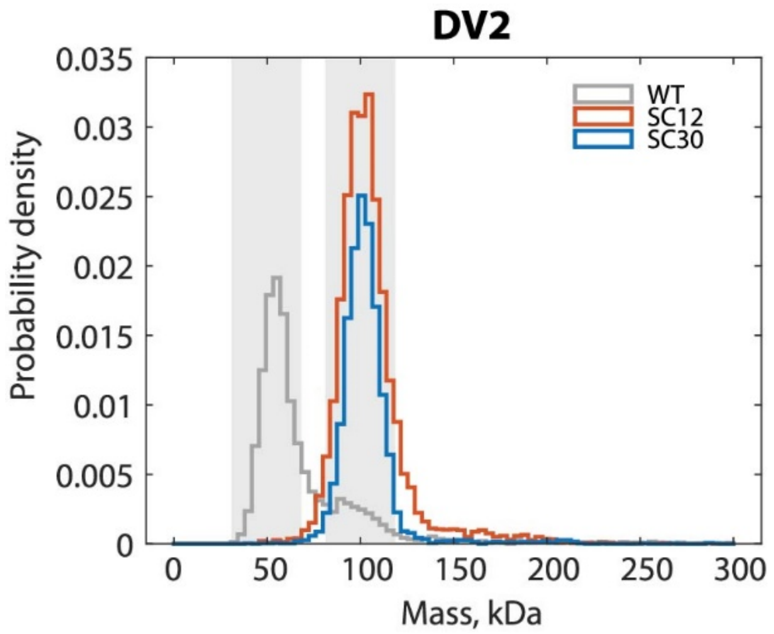

Figure S4. Oligomeric state of DENV2 sE as determined by mass photometry. 50 nM DENV2 sE WT (grey), SC12 (orange) and SC30 (blue) was used for the experiment. Histograms show shaded regions surrounding monomer mass (~50 kDa) and dimer mass (100 kDa).

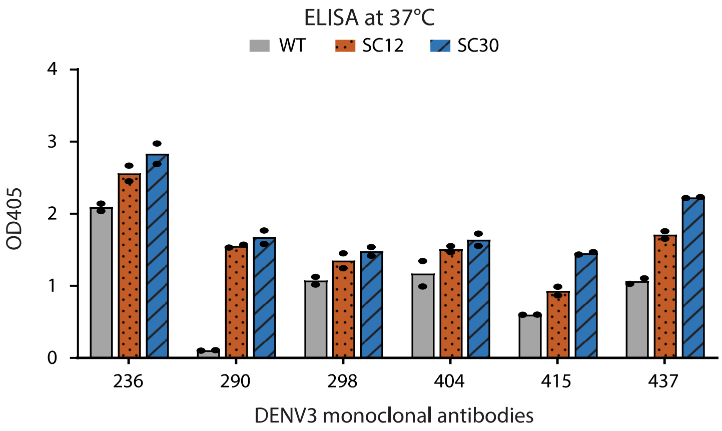

Figure S5. Single-point ELISA at 37°C between DENV3 sE proteins and DENV3 type-specific mAbs. 45 nM sE WT (grey), SC12 (orange, dotted) and SC30 (blue, striped) was immobilized on Nickel ELISA plate and incubated with 2 ng/µL (~13 nM) IgG, bars represent mean of technical duplicates.
